# A New Article for the Treatment of Tape-Worm

**Published:** 1852-11

**Authors:** 


*A New Article for the Treatment of Tape-Worm.* By ALFRED S. CASTLEMAN, M. D.

In 1844, I was at the "taking of a bee tree," at which a delicate child, of some ten or twelve years, ate freely of the honey comb, from which most of the honey had been pressed. Next day the child was purged, and sticking to the wax, which passed almost unchanged, were many pieces of tape-worm varying from half an inch to ten inches in length.

Since then, I have had opportunity of trying the honey in three cases, in which there was *no doubt* of the existence of the worm. In two of them it was entirely successful, in the third it failed; in each of them the patient was directed to eat, at short intervals, for twenty-four hours, as much new honey in the comb as the stomach would comfortably bear (otherwise fasting.) A cathartic was then administered, and the worm was ejected in short pieces, but alive.

A *country* practitioner might pass a lifetime in business and not meet with cases enough of this kind to prove or disprove the claims of any article to the title of remedy, but where a thing so simple, so easily obtained, so harmless in action, has proven successful in three of the only four cases in which it has been used, I feel that I am excusable in calling the profession to assist in ascertaining whether it has any claims to our confidence. I do not know that it is a remedy, nor do I recommend it as such; I barely state the facts which have come under my observation, and ask assistance in ascertaining whether they are worth preserving.

Delafield, Wis., August, 1852.—*North Western Med. and Surg. Jour.*

